# The therapeutic efficacy of Xuanfei Baidu Formula combined with conventional drug in the treatment of coronavirus disease 2019: A protocol for systematic review and meta-analysis

**DOI:** 10.1097/MD.0000000000024129

**Published:** 2021-01-22

**Authors:** Fan Li, Yajuan Li, Jingxia Zhang, Shasha Li, Ajuan Mao, Chongbo Zhao, Weifeng Wang, Fang Li

**Affiliations:** a Shaanxi Academy of Traditional Chinese Medicine, Xi’an, Shaanxi, China; b Pharmacy College, Shaanxi University of Chinese Medicine, Xianyang.

**Keywords:** conventional drug, corona virus disease 2019, meta-analysis, protocol, systematic review, Xuanfei Baidu Formula

## Abstract

**Background:**

Coronavirus disease 2019 (COVID-19) is a new acute espiratory infectious disease that has been in a public health emergency of international concern. In China, the combination of Xuanfei Baidu Formula (XBF) and conventional drug is used in the clinical treatment of patients with new coronary pneumonia, However, there is no comprehensive and systematic evidence on the effectiveness and safety of XBF.

**Materials and Methods:**

We search for research in PubMed, China National Knowledge Infrastructure, Wan-fang Database, China Biomedical Database, and Chinese Science Citation Database. For “Xuanfei Baidu Formula” and “COVID-19,” we screened suitable articles without language restrictions on keywords, Review Manager 5.3 and STATA 14.2 software was used for the data analysis.

**Results:**

The systematic review and meta-analysis will evaluate the efficacy and safety of XBF combined with conventional drug in the treatment of COVID-19.

**Conclusion:**

We will provide evidence of XBF for the treatment on COVID-19 patients.

**INPLASY Registration number:**

INPLASY2020120011

## 1 Introduction

Corona Virus Disease 2019 (COVID-19) is an RNA virus caused respiratory disease, which has posed an enormous threat to public health.^[1]^ its long incubation period, strong contagiousness, the main transmission route is Respiratory droplet, also, it could be transmitted through person-to-person contacts by asymptomatic carriers.^[2]^ According to statistics from the World Health Organization, as of Dec 3, 2020, This brings the cumulative numbers to over 61.8 million reported cases and 1.4 million deaths globally since the start of the pandemic.^[3]^ There is no effective way to control the COVID-19 and no vaccine is currently available.^[4]^ The treatment is only symptomatic, Therefore, the effective prevention and treatment of COVID-19 are a very urgent task.

Traditional Chinese medicine (TCM) has been proven effective for COVID-19 treatment.^[5]^ Xuanfei Baidu Formula was recommended as the treatment agent in the 8th edition of the "Diagnosis and Treatment Scheme for New Coronavirus Infected Pneumonia.^[6]^ Xiong Wuzhong et al recent study showed that Xuanfei Baidu Formula (XBF) is safe and effective for COVID-19 and can significantly improve COVID-19 patient's clinical symptoms, increase the number of white blood cells and lymphocytes to improve immunity, and also significantly reduce C-reactive protein and erythrocyte sedimentation rate to play an anti-inflammatory effect.^[7]^ Because of comprehensive and systematic evidence, we will collect all randomized controlled trials on XBF in the treatment of COVID-19, and present a meta-analysis protocol and systematic reviews.

## 2 Methods and program

The protocol has been registered on the International prospective register of systematic review (INPLASY), the registration number is INPLASY2020120011 (DOI: 10.37766/inplasy2020.12.0011). The protocol followed Preferred Reporting Items for Systematic review and Meta-Analysis Protocols (PRISMA-P) guidelines.^[8]^

### 2.1 Literature search strategy

We search for research in PubMed, China National Knowledge Infrastructure, Wan-fang Database, China Biomedical Database, and Chinese Science Citation Database. Exemplary search strategy of PubMed is listed in Table 1. Full-text review was performed while the title/abstract thought to be thematic. The job above was executed by 2 investigators independently. The conflicts were resolved by consensus and discussion.

**Table 1:** PUBMED search strategy.

Number	Search terms
1	“randomized controlled trial” [Publication Type] OR “controlled clinical trial” [Publication Type] OR “Single-Blind Method” [Text Word] OR “random allocation” [Text Word] OR “RCT” [Text Word] OR “RCTs” [Text Word]
2	“Xuanfei Baidu Formula” [All Fields] OR “Xuanfei Baidu Decoction” [All Fields] OR “XuanfeiBaidu Formula” [All Fields] OR “XFB” [All Fields] OR “XFB Formula” [All Fields]
3	“COVID-19” [Title/Abstract] OR “coronavirus disease 2019” [Title/Abstract] OR“2019 novel coronavirus disease” [Title/Abstract] OR “2019-nCoV ” [Title/Abstract] OR “SARSCov2 ” [Title/Abstract]
4	#1 AND #2 AND #3

### 2.2 Inclusion criteria

#### 2.2.1 Study design

The study trials were randomized controlled trials, participants age of 18 to 75 years old.

#### 2.2.2 Diagnostic criteria

The specific IgM and IgG antibodies against SARS-COV-2 were positive in serum test.

#### 2.2.3 Intervention group

The treatment group was given XBF on the basis of conventional drug treatment. Control group: only conventional drug treatment.

#### 2.2.4 Main outcomes

Total efficacy, relief time of main symptoms (such as fever, cough, myalgia, or fatigue), relief time of other symptoms (such as headache, dizziness, diarrhea, nausea and so on.) Additional outcomes: leukocyte, lymphocyte, C-reactive protein, adverse events.

### 2.3 Exclusion criteria

**2.3.1** Some reviews, animal experiments, case report and comments et al. were thought to be unrelated with the topic.

**2.3.2** Experiments without control or Diagnostic criteria statement was ambiguous.

**2.3.3** The intervention of COVID-19 patients was not based on XBF treatment.

**Figure 1:**
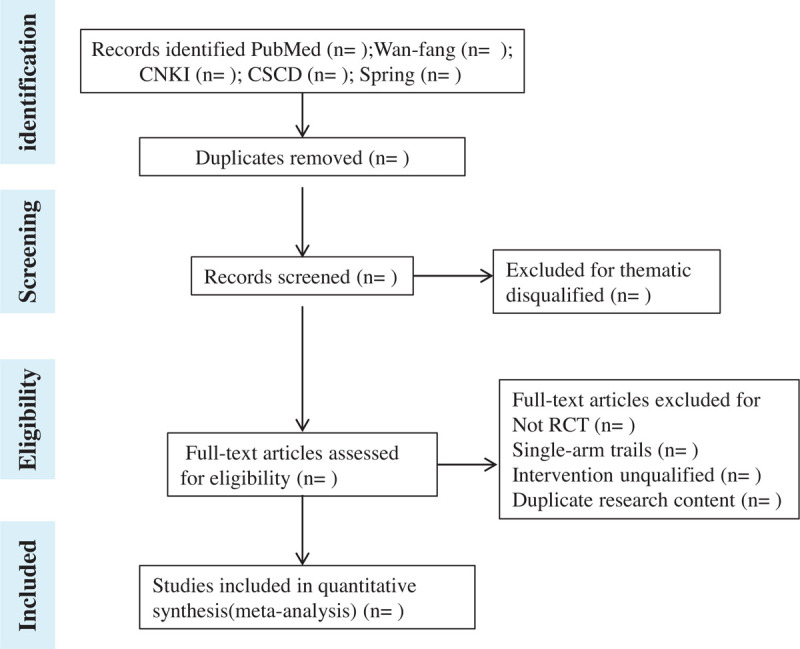
Process of study screening.

The full screening process is shown in PRISMA flow chart (Fig. 1).

### 2.4 Data extraction and quality assessment

Summarize the searched literatures, including basic characteristics, basic information, intervention measures, Outcome indicators. For example, author, publication time, number of samples in each group, age, ratio of male to female, drugs, time used in treatment, and so on. Then extracted data were assessed for Cochrane bias risk by 2 investigators independently.^[9]^ as follows: random sequence generation (selection bias), allocation concealment (selection bias), blinding of participants and personnel (performance bias), blinding of outcome assessment (detection bias), incomplete outcome data (attrition bias), selective reporting (reporting bias) and other bias. Each term was judged with 3 levels-Low risk, High risk, Unclear risk” bias, If there are disagreements during the evaluation process, it will be resolved through discussion with a third investigator.

### 2.5 Data analysis

Data analysis was performed using Review Manager 5.3 (Cochrane Collaboration) and STATA 16.0 software. Outcome measures such as the total effective rate, the disappearance rate of main clinical features and the disappearance rate of minor symptoms were regarded as dichotomous variables and presented as the odds ratio (OR) with 95% confidence intervals (95% CI), and the risk ratio (OR) with 95% confidence intervals (95% CI). Contents of inflammatory cytokines were continuous variables that presented as the mean difference (MD) with 95% CI, *Q* statistic and *I*^2^ tests were applied to assess the heterogeneity among studies.

When *I*^2^≤ 25%, the data is considered homogeneous. A fixed-effects model was used to analyze data with low heterogeneity (25%≤ *I*^2^≤ 50%) and data with high heterogeneity (*P* < .1 or *I*^2^ > 50%) was estimated using random-effects model. Potential publication bias was revealed by funnel plots.^[10,11]^

## 3 Discussion

We study to evaluate the clinical effect and safety of XBF on patients infected with COVID-19. With the advent of winter, many experts predict that the epidemic caused by COVID-19 will once again erupt globally.^[12,13]^ Traditional Chinese medicine has a history of more than 2000 years in the prevention and treatment of epidemics and plagues,^[14]^ In light of its experience treating SARS and H1N1 influenza, in the fight against COVID-19, China achieved effect. Network pharmacology and molecular docking studies of XBF show that it has effect in the treatment of COVID-19 patients, by regulating key targets such as IL6, MAPK3, MAPK1, IL1β, CCL2, EGFR.^[15]^ This study aims to evaluate the effectiveness and safety of XBF in the treatment of COVID-19, and to provide ways for the treatment of COVID-19.

## Author contributions

**Methodology**: Fan Li.

**Project administration**: Fan Li.

**Software**: Jingxia Zhang, Shasha Li.

**Supervision**: Fang Li.

**Visualization**: Yajuan Li, Aj Mao.

**Writing – original draft**: Fan Li, Chongbo Zhao.

**Writing – review & editing**: Fang Li, Weifeng Wang

## References

[1] Gennaro FD Pizzol D Marotta C Coronavirus diseases (COVID-19) current status and future perspectives: a narrative review. Int J Environ Res Public Health 2020;17:2690. 10.3390/ijerph17082690 PMC7215977 32295188

[2] Yang YS Peng FJ Wang RS The deadly coronaviruses: the 2003 SARS pandemic and the 2020 novel coronavirus epidemic in China. J Autoimmun 2020;109. 10.1016/j.jaut.2020.102434 PMC7126544 32143990

[3] Coronavirus disease (COVID-19). World Health Organization, Available at: https://www.who.int/publications/m/item/weekly-epidemiological-update---1-december-2020.

[4] Wang HL Wang SC Yu KJ COVID-19 infection epidemic: the medical management strategies in Heilongjiang Province, China. Critical Care 2020;24 (1.):10.1186/s13054-020-2832-8 PMC7081588 32188482

[5] Liu M Gao Y Yuan Y Efficacy and Safety of Integrated Traditional Chinese and Western Medicine for Corona Virus Disease 2019 (COVID-19): a systematic review and meta-analysis. Pharmacol Res 2020;158:104896. 32438037 10.1016/j.phrs.2020.104896 PMC7211759

[6] National Health Commission of the People's Republic of China. The guideline on diagnosis and treatment of coronavirus disease 2019 (Revised 8th version). 10.21147/j.issn.1000-9604.2019.02.02 PMC6513740 31156298

[7] Xiong WZ Wang G Du J Efficacy of herbal medicine (Xuanfei Baidu decoction) combined withconventional drug in treating COVID-19: a pilot randomizedclinical trial. Integr Med Res 2020;9:100489. 32874913 10.1016/j.imr.2020.100489 PMC7452296

[8] Shamseer L Moher D Clarke M Preferred reporting items for systematic review and meta-analysis protocols (PRISMA-P) 2015: elaboration and explanation. BMJ (Clinical research ed) 2015;350:g7647. 10.1136/bmj.g7647 25555855

[9] Deeks JJ Higgins JPT Altman DG Green S Cochrane Handbook for Systematic Reviews of Interventions Version 5.1.0. Available at: http://training.cochrane.org/handbook 2011.

[10] Zou JB Zhang XF Wang J The therapeutic efficacy of danhong injection combined with percutaneous coronary intervention in acute coronary syndrome: a systematic review and meta-analysis. Front Pharm 2018;9:550. 10.3389/fphar.2018.00550 PMC5994407 29915535

[11] Higgins JP Thompson SG Deeks JJ Measuring inconsistency in meta-analyses. BMJ (Clinical research ed) 2003;327:557–60. 10.1136/bmj.327.7414.557 PMC192859 12958120

[12] Fazzini M Baresi C Bisci C Preliminary analysis of relationships between COVID19 and climate morphology, and urbanization in the Lombardy region (Northern Italy). Int J Environ Res Public Health 2020;17:6955. 10.3390/ijerph17196955 PMC7579304 32977546

[13] Meo SA Abukhalaf AA Alomar AA Effect of heat and humidity on the incidence and mortality due to COVID-19 pandemic in European countries. Eur Rev Med Pharmacol Sci 2020;24:9216–25. 32965017 10.26355/eurrev_202009_22874

[14] Wang JJ Qi FH Traditional Chinese medicine to treat COVID-19: the importance of evidence-based research. Drug Discov Ther 2020;14:149–50. 32669523 10.5582/ddt.2020.03054

[15] Wang H Song HX Wang DF Potential mechanism of Xuanfei Baidu Formula in treating new coronavirus pneumonia based on network pharmacology and molecular docking. J Hainan Medical University 2020;26:1361–72.

